# Daily feeding and protein metabolism rhythms in Senegalese sole post-larvae

**DOI:** 10.1242/bio.021642

**Published:** 2016-11-28

**Authors:** Carmen Navarro-Guillén, Manuel Yúfera, Sofia Engrola

**Affiliations:** 1 Instituto de Ciencias Marinas de Andalucía (ICMAN-CSIC), Apartado Oficial, Cádiz, Puerto Real 11519, Spain; 2 Centro de Ciências do Mar (CCMAR), Edifício 7, Universidade do Algarve, Campus de Gambelas, Faro 8005-139, Portugal

**Keywords:** Daily feeding pattern, Feed intake, Post-larvae, Protein metabolism, *Solea senegalensis*

## Abstract

Fish hatcheries must adapt larval feeding protocols to feeding behavior and metabolism patterns to obtain more efficient feed utilization. Fish larvae exhibit daily ingesting rhythms rather than ingesting food continuously throughout the day. The aim of this study was to determine the daily patterns of feed intake, protein digestibility, protein retention and catabolism in Senegalese sole post-larvae (*Solea senegalensis*; 33 days post-hatching) using ^14^C-labeled *Artemia* protein and incubation in metabolic chambers. Sole post-larvae were fed at 09:00, 15:00, 21:00, 03:00 and 09:00+1 day; and those fed at 09:00, 21:00, 03:00 and 09:00+1 day showed significantly higher feed intake than post-larvae fed at 15:00 h (*P*=0.000). Digestibility and evacuation rate of ingested protein did not change during the whole cycle (*P*=0.114); however, post-larvae fed at 21:00 and 03:00 h showed the significantly highest protein retention efficiency and lowest catabolism (*P*=0.002). Therefore, results confirm the existence of daily rhythmicity in feeding activity and in the utilization of the ingested nutrients in Senegalese sole post-larvae.

## Abstract (precis)

**Summary:** This manuscript provides new knowledge regarding Senegalese sole post-larvae daily feeding behavior and protein metabolism, which are essential for the adequate development of feeding strategies for this species.

## INTRODUCTION

In order to obtain high quality fish, larvae need to be efficient in ingesting and processing food. Thus, marine fish hatcheries need to adapt larval feeding protocols to incorporate feeding behavior and metabolism patterns in order to trim costs and increase their revenue. Feeding frequency and amount of food per meal are key parameters that influence digestion and nutrient absorption, and consequently fish growth performance. Nevertheless, our knowledge of the effect of feeding regimes in fish larvae growth is still scarce and limited to a few species. Comparable growth rates were found in the Amazonian ornamental fish *Pyrrhulina brevis* fed two and four meals per day (Veras et al., 2016). In contrast, the larvae of large yellow croacker (*Pseudosciaena crocea*) (Xie et al., 2011) fed frequently had improved growth and survival when compared to fish fed less frequently. Likewise, Senegalese sole (*Solea senegalensis*) pulse-fed during the pre-weaning period showed a better growth performance after weaning than sole fed twice daily, suggesting that feeding frequency during early larval stages has an impact in later ages (Engrola et al., 2005).

Feed intake assessment is pivotal for any nutritional study, as it is closely associated with gut transit time and digestive and absorptive efficiency of dietary nutrients (Conceição et al., 2007a). Fish larvae do not display constant food ingestion under natural or laboratory conditions, instead they exhibit daily feeding rhythms (Kotani and Fushimi, 2011; Mata-Sotres et al., 2015; Navarro-Guillén et al., 2015). Most animals are active either during the day or at night, and different species have acquired behavioral patterns under the influence of relatively stable selective forces. The light/dark cycle is recognized as the main factor that entrains biological rhythms in teleost fishes through its influence on feeding physiology and gene expression (Montoya et al., 2010; Del Pozo et al., 2012; Martín-Robles et al., 2012, Vera et al., 2013; Mata-Sotres et al., 2015). Circadian rhythms, in which are included feeding rhythms, are generated and regulated by transcriptional-translational feedback loops of a group of circadian clock genes and their derived proteins (Dunlap, 1999; Albrecht, 2002; Mata-Sotres et al., 2015). The effect of both photoperiod and the feeding regime on clock gene expression has been described for several species, such as gilt-head seabream (*Sparus aurata*) (Vera et al., 2013; Paredes et al., 2014; Mata-Sotres et al., 2015), Atlantic salmon (*Salmo salar*) (Davie et al., 2009), rainbow trout (*Oncorhynchus mykiss*) (Davie et al., 2011), European seabass (*Dicentrarchus labrax*) (Del Pozo et al., 2012; Herrero and Lepesant, 2014) and Senegalese sole (*Solea senegalensis*) (Martín-Robles et al., 2012, 2013). However, in some species, the temporal pattern of behavior may change during ontogeny. A shift from diurnal to nocturnal feeding behavior, concomitant with the change from pelagic to benthic habitats in early stages, has been described in some flatfishes such as tongue sole (*Cynoglossus semilaevis*) (Ma et al., 2006) and Senegalese sole (Cañavate et al., 2006; Navarro-Guillén et al., 2015).

Senegalese sole (*Solea senegalensis* Kaup 1858) is considered a promising candidate for marine aquaculture in the south of Europe due to its high price, market demand and high growth potential (Imsland et al., 2003; Morais et al., 2016). This species has a complex metamorphosis with marked anatomical changes. After metamorphosis, the shift from a pelagic to a benthic habitat implies significant changes in feeding habits as well as in digestive physiology (Ribeiro et al., 1999; Fernández-Díaz et al., 2001; Engrola et al., 2009b; Navarro-Guillén et al., 2015). Interestingly, there is no reversal in the phase of clock gene rhythms between pre- and post-metamorphic individuals that would be coincident with the switch from diurnal to nocturnal feeding and locomotor activity. Whether specialized central pacemakers dictate the phase of locomotor activity or this control is exerted outside of the core clock mechanism remains to be elucidated (Martín-Robles et al., 2013). Senegalese sole larvae has a high digestive capacity for digesting live prey from the onset of exogenous feeding, which is reflected in their high growth potential (Parra and Yúfera, 2001; Conceição et al., 2007b, Engrola et al., 2009a). Thus, early weaning strategies in Senegalese sole are based on *Artemia* replacement, since the inert diet alone is not sufficient to sustain fish larvae development. A moderate replacement of live prey by inert diet in early stages has been shown to promote larval growth at later stages (Engrola et al., 2009b). However, above a replacement threshold, protein digestibility and retention diminish and larval growth is depressed (Engrola et al., 2010).

Several studies have determined the fate of ingested protein in fish larvae (Rønnestad et al., 2001; Morais et al., 2004a; Conceição et al., 2007a; Engrola et al., 2009a, 2010; Richard et al., 2015; Canada et al., 2016 preprint; Rocha et al., 2016). Nonetheless, quantifying feed intake based on a single meal, particularly after a pre-conditioning period under starvation conditions, may not give representative results, given that at this time the appetite may be at its maximum. To optimize feeding protocols, a broader knowledge of circadian feeding behavior and digestive metabolism patterns is needed. The potential effect of the daily feeding rhythm on digestive and absorption efficiency remains unknown in fish larvae, and it represents a gap that is necessary to fill in order to improve growth performance. With this aim, this study analyses the feed intake and protein metabolism of Senegalese sole post-larvae over 24 h.

## RESULTS

### *Artemia* intake

**Fig. 1.**
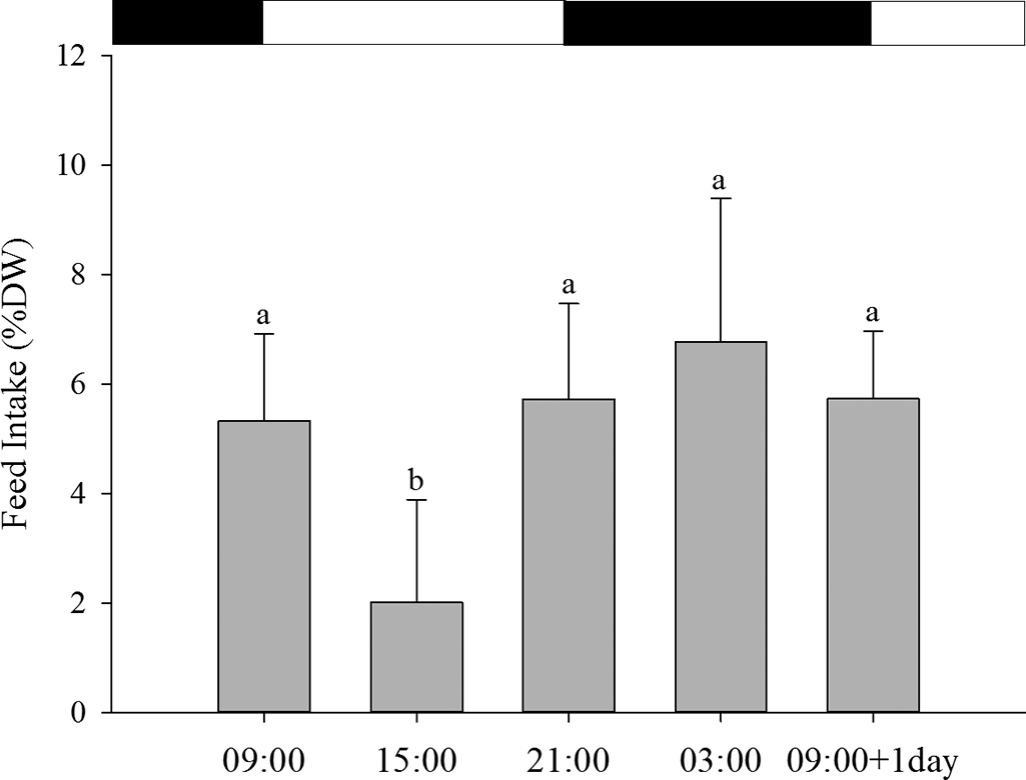
**Feed intake of 33 dph Senegalese sole post-larvae at the different time points.** 09:00 h (*n*=10), 15:00 h (*n*=11), 21:00 h (*n*=12), 03:00 h (*n*=12) and 09:00 h+1 d (*n*=9). Results are shown as means±s.d. Letters indicate significant differences between time points (one-way ANOVA, *P*=0.000). Photoperiod is indicated by the horizontal bars; light period (white bars) and dark period (black bars).

Post-larvae ate over the whole 24-h cycle but feed intake levels were significantly different depending on the time of the day (*P*=0.000; Fig. 1). Higher feed intakes were recorded at 09:00, 21:00 and 03:00 h than at 15:00 h. The number of *Artemia* consumed per sole post-larvae during a 30 min period was (mean±s.d.) 72.46±21.79 [5.32±1.60% larval body dry weight (DW)] at 09:00 h; 27.43±25.48 (2.01±1.87% DW) at 15:00 h; 77.87±23.82 (5.72±1.75% DW) at 21:00 h; 92.14±35.77 (6.76±2.63% DW) at 03:00 h; and 78.00±16.85 (6.76±2.63% DW) at 09:00 h+1 d.

### *Artemia* protein digestibility and metabolism

**Fig. 2.**
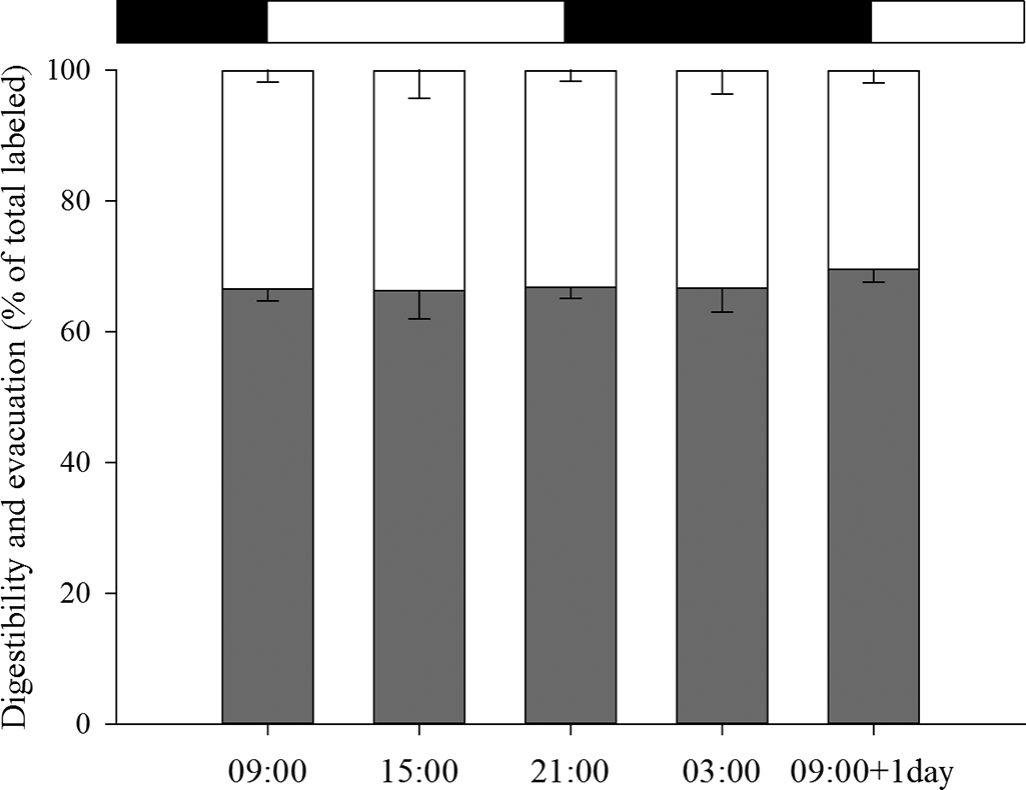
***Artemia* protein digestibility and evacuation.** *Artemia* protein digestibility (gray bars; % of radiolabel in the sole body and CO_2 trap_ in relation to the total radiolabel fed) and *Artemia* protein evacuation (white bars; % of radiolabel in sea water in relation to the total radiolabel fed) in 33 dph Senegalese sole post-larvae at the different time points and after 18 h of incubation. Results are shown as means±s.d. No statistical differences were found in protein digestibility and evacuation between sampling points (one-way ANOVA, *P*=0.114). Photoperiod is indicated by the horizontal bars; light period (white bars) and dark period (black bars).

Protein digestibility values ranged between 66.34±4.34% at 15:00 h and 69.57±1.93% of total labeled at 09:00 h+1 d, and no statistical differences were observed between meals (*P*=0.114; Fig. 2). Similarly, there were no statistical differences in the proportion of protein evacuated as a function of the feeding time, with values ranging between 30.43±1.93% (at 09:00 h+1 d) and 33.66±4.34% of total labeled (at 15:00 h) (*P*=0.114).

**Fig. 3.**
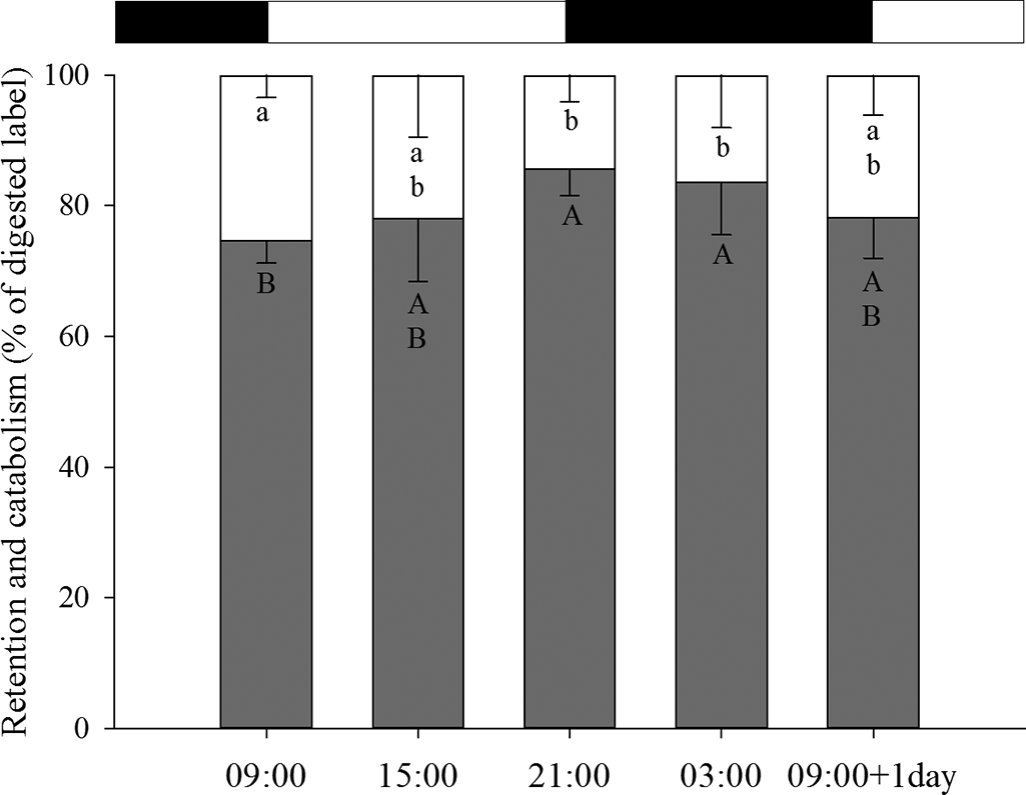
***Artemia* protein retention and catabolism.** *Artemia* protein retention (gray bars; % of radiolabel in the sole in relation to digested label), and catabolism (white bars; % of radiolabel in the metabolic trap in relation to digested label) in 33 dph Senegalese sole post-larvae at the different time points and after 18 h of incubation. Values are shown as means±s.d. Different letters indicate statistically significant differences (one-way ANOVA, *P*=0.002). Photoperiod is indicated by the horizontal bars; light period (white bars) and dark period (black bars).

However, sole post-larvae fed at 21: 00 h and 03:00 h presented the highest protein retention efficiencies (% of absorbed label; 85.64±4.04 and 83.61±8.07, respectively) (Fig. 3), while the lowest protein retention efficiency was measured at 09:00 h (74.69±3.45% of absorbed label). At 15:00 h and 09:00 h+1 d retention efficiencies were 78.05±9.59% and 78.10±6.14% of absorbed label, respectively (*P*=0.002). In parallel, post-larvae fed at 09:00 h showed a significantly higher catabolism (25.31±3.45% of absorbed label) than those fed at 21:00 h and 03:00 h (14.36±4.04 and 16.39±8.07%, respectively). Catabolism efficiencies were 21.95±9.59% and 21.90±6.14% of absorbed label at 15:00 h and 09:00 h+1 d respectively (*P*=0.002).

## DISCUSSION

Feed intake measurements are pivotal for understanding larval nutrition and physiology. Several techniques have been used to determine feed intake in larval fish, such as direct observation of prey within the gut, declining of prey concentration in the rearing tanks, image analysis and prey labeling with fluorescent markers, inert metal oxides or with radiolabeled tracers (Yúfera et al., 1995; Olsen et al., 2000; Parra and Yúfera, 2000; Cañavate et al., 2006; Conceição et al., 2007a; Engrola et al., 2009a, 2010). The radiolabel methodology is the only technique that combines feed intake and metabolism in a single approach (Morais et al., 2004a). The present results show that sole post-larvae change their feed intake during the daily cycle. Post-larvae fed at 15:00 h showed significantly lower feed intake than the remaining sampling points. The highest feed intake recorded (at 03:00 h) was 3.35-fold higher than the lowest (at 15:00 h). The amount of *Artemia* offered was the same in all feeding times, so the observed differences may reflect variations in the appetite control during the day. Proteins and free amino acids (FAA) contained in the chyme have been identified as signals initiating the release of the digestive hormone cholecystokinin (CCK), which is a key factor causing the release of pancreatic digestive enzymes as trypsin into the gut lumen and is also involved in appetite regulation (Koven et al., 2002; Rønnestad et al., 2013; Tillner et al., 2013). Tillner et al. (2013) described a regulation system mediated by a feedback mechanism between gut CCK and tryptic enzyme activity in Atlantic cod larvae (*Gadus morhua*), and later in European seabass (*Dicentrarchus labrax*) (Tillner et al., 2014). Navarro-Guillén et al. (2017) described this CCK-trypsin regulatory loop in Senegalese sole larvae. Additionally, results also suggested the implication of the hormone CCK in appetite regulation in Senegalese sole, with CCK levels tending to be lower when larvae had been fed and higher when food was not available. Nevertheless, these trends were less prominent in later post-larvae (32 dph) and further studies using techniques that permit more detailed analysis of paracrine effects also linked with neural signaling pathways should be explored to get a better knowledge of Senegalese sole appetite regulation, since appetite is definitely a key factor to understand feed intake and satiety events.

The gut content also gives an estimation of feed intake, but evacuation rates are needed to obtain accurate ingestion values; and gut residence time is difficult to determine in early fish larval stages. Navarro-Guillén et al. (2015) found that gut content level in Senegalese sole early larvae under continuous feeding follows a clear daily rhythm, filling the gut during the day and evacuating during the night. In contrast, in post-larvae the gut content was constant over the daily cycle, although a trend of higher gut content was found at dark hours (Navarro-Guillén et al., 2015, 2017). Therefore, together with the present data, results confirm that one-month-old post-larvae are in the transition phase from a diurnal to a preferentially nocturnal feeding, as described in juveniles and adults (Cañavate et al., 2006; Navarro et al., 2009).

*Artemia* protein digestibility in the present study ranged from 66.34% (15:00 h) to 69.57% of total labeled (09:00 h+1 d). Similar values have been reported in 35 dph sole post-larvae using a similar approach of one-single meal after an unfed preconditioning (Morais et al., 2004b; Engrola et al., 2009a). In the present study, no differences were observed in *Artemia* protein digestibility and the values are within those expected for their age, since sole post-larvae have a fully developed and functional digestive tract (Conceição et al., 2007b). The higher content of soluble nitrogen in *Artemia* (54% of total nitrogen) (Carvalho et al., 2003) may explain the higher *Artemia* protein digestibility usually observed. It could be expected that the feeding rate had some impact on the digestive intensity. A higher food intake may imply a faster gut transit and consequently, a shorter time available for efficient digestion and absorption of amino acids. In addition, an excessively fast gut transit may limit the appropriate recovery of the enzymes in the chyme (Rønnestad et al., 2013); nevertheless, in our study the digestive capacity was not influenced by feeding rate. In general, the digestion and subsequent absorption and retention processes seem to be quite fast throughout larval development in Senegalese sole, given that 3 h after feeding the amount of label found in the body tissues was stabilized to around 45% of total label (Morais et al., 2004b). In Senegalese sole post-larvae, trypsin activity showed similar values between 12:00 h and 06:00 h (Navarro-Guillén et al., 2015) and although the tryptic activity declined at the end of the dark period, it sharply increased again just after the onset of the light period. This daily pattern may explain the absence of differences in *Artemia* protein digestibility observed in the present study.

Similarly, modifications in feeding regimes, like feeding frequency, may alter the digestion efficiency. Turbot larvae fed three times a day and rotifers reacted to the first two meals with immediate trypsinogen secretion, but not to the third meal (Rønnestad et al., 2013). Senegalese sole post-larvae reared in continuous feeding showed a sinusoidal feeding pattern with four feeding peaks within the day, and the amount of food ingested during the night (dark) period was statistically higher when compared to the light period (Navarro-Guillén et al., 2017). Senegalese sole post-larvae re-fed with a second meal two hours after the first one, and showed a higher protein digestibility than post-larvae fed one single meal, suggesting that the second meal might have stimulated the enzyme secretion as a reaction to feed intake and increased the digestibility of the first meal (Engrola et al., 2009a). In the present study, no differences were found in *Artemia* protein digestibility. The absence of differences may indicate that maximum protein digestibility was reached, and that during the 6 h intervals fish were able to complete digestion.

In general, predominantly nocturnal species show better food utilization and improved growth when fed an imposed regime at night rather than at daytime, and vice versa (Azzaydi et al., 2000; Hossain et al., 2001; Marinho et al., 2014). This statement agree with results presented in the present study, since although there were no statistical differences in protein digestibility during the day, the highest protein retention capacities were reached in post-larvae fed at nighttime (21:00 h and 03:00 h). Conversely, Engrola et al. (2002) and Marinho et al. (2014) found that juvenile sole, for a period of their life cycle, appear to use dietary protein more efficiently for somatic growth under a diurnal rather than under a nocturnal feeding regime. This suggests that at least during a time-window in the juvenile rearing, a diurnal feeding regime might be more effective in the production of this species. Research into other species found a marked effect of feeding time on growth performance. For European seabass, which show phase inversion in their feeding rhythms on a seasonal basis, results revealed that feeding seabass by night, in winter, may result in improved growth performance. Although the flexibility of seabass feeding habits enabled fish to adapt to diurnal feeding, the cost of adaptation to feeding out of phase with the natural feeding rhythms seems to reduce growth (Azzaydi et al., 2000). Similarly, in the African catfish (*Clarias gariepinus*), the growth rates of fish fed continuously or during nighttime following their feed demand were significantly higher with lowest food conversion ratios and food wastage (Hossain et al., 2001). In contrast, Heilman and Spieler (1999) reported improved growth and higher feed efficiency in the Florida pompano (*Trachinotus carolinus*) when the time of food supply was out of phase with the preferred feeding time. This variety in the results between species indicates that different feeding times cause distinct realignments of several circadian rhythms, which could lead to profound variations on metabolism (Spieler, 2001). Therefore, studies for understanding species nutrition and physiology are crucial for the correct design of feeding protocols, and consequently, the proper fish development.

In conclusion, this study contributes to understanding how Senegalese sole post-larvae utilize protein during a daily cycle and how feed intake may influence protein metabolism. Post-larvae showed a higher *Artemia* intake at 09:00 h, 21:00 h and 03:00 h and 09:00 h+1 d compared to other feeding times, indicating the existence of daily rhythmicity. Regarding protein metabolism, post-larvae showed constant digestibility values over the whole day, although the highest protein retention efficiency and lowest catabolism were observed at nighttime (21:00 h and 03:00 h), thus confirming also the existence of daily rhythmicity in the utilization of the ingested nutrients.

## MATERIAL AND METHODS

The experiments were carried out in compliance with the Guidelines of the European Union Council (2010/63/EU) and Spanish legislation for the use of laboratory animals, with approval of the Bioethics Committee of the Spanish National Research Council for project EFISHDIGEST (AGL2014-5288-R). CCMAR facilities and their staff are certified to house and conduct experiments with live animals (‘group-1’ license by the ‘Direção Geral de Veterinaria’, Ministry of Agriculture, Rural Development and Fisheries of Portugal).

### Feed intake, digestion and retention of radiolabeled *Artemia* protein by Senegalese sole post-larvae

#### Senegalese sole post-larval daily feeding rhythm

On the day prior to the metabolic assays, 60 post-larvae of Senegalese sole were transferred and split between five small containers (each container corresponding to one sampling point with 12 post-larvae container^−1^) with clean seawater in the nutrient flux laboratory. The post-larvae were acclimated overnight and fed with unlabeled *Artemia* metanauplii (cold) in order to avoid any fasting effect. Photoperiod in the nutrient flux laboratory was 12 h light:12 h dark (lights turned on and off at 9:00 h and 21:00 h, respectively). Trials were conducted every 6 h for a 24 h period: 09:00 h, 15:00 h, 21:00 h, 03:00 h and 09:00 h +1 d (day). The time between protein metabolism determinations was based on previous results (Navarro-Guillén et al., 2017) which described a rhythmicity of 6 h between meals when fish were fed *ad libitum*.

During the trials, *Artemia* concentration was determined by counting as described below and 6000 radiolabeled *Artemia* metanauplii (hot) were added to the corresponding container and the same quantity of unlabeled *Artemia* metanauplii (cold) were offered to post-larvae in the remaining containers. Post-larvae were allowed to feed for 30 min, since this period is a suitable trade-off between the time necessary for a complete meal while avoiding significant losses by larvae catabolism (Engrola et al., 2009a; Mai et al., 2009). After this period, 12 post-larvae fed radiolabeled *Artemia* (*n*=12) were individually rinsed twice in clean seawater and individually incubated for 18 h in vials containing 6 ml of seawater in a sealed system, linked up to a CO_2_ metabolic trap (5 ml 0.5 mol l^−1^ KOH) (Rønnestad et al., 2001). The remaining containers were carefully cleaned in order to remove the uneaten unlabeled *Artemia* (cold). This protocol was carried out at each sampling point along the 24 h cycle.

After acidification (with 1 ml 0.1 M HCl) of incubation water vials, the fraction of the label that was catabolized by the post-larvae and became entrapped in seawater by conversion to HCO^−3^ was recovered in the metabolic traps as ^14^CO_2_ that diffused out of the water. Finally, the label remaining in the water corresponds to *Artemia* protein that was evacuated. In order to determine feed intake and protein retention after incubation period, whole post-larvae were individually dissolved in 0.75 ml of an aqueous based solubilizer (Solvable™, PerkinElmer, USA). Equally, radiolabeled *Artemia* (hot) samples were also dissolved adding 1 ml of solubilizer. Samples were maintained at 40°C for 48 h.

#### *Artemia* [U-^14^C] labeling

*Artemia* cysts were incubated for 24 h in a plastic cylindrical-conical flask, under standard conditions (Van Stappen, 1996). The newly hatched *Artemia* nauplii were harvested and washed on 150-μm plankton net and transferred to clean seawater. Their concentration was determined by counting the nauplii under a binocular microscope. For *Artemia* metanauplii [U-^14^C] labeling, newly hatched nauplii (200 nauplii ml^−1^) were stocked in a single cylindrical glass container with 150 ml of seawater vigorously aerated at 28°C. *Artemia* nauplii were kept for 6 h, which is approximately the time needed to reach their first feeding stage (Van Stappen, 1996). After that period, *Artemia* nauplii were enriched with a [U-^14^C] uniformly labeled protein hydrolysate (3.7 MBq ml^−1^, American Radiolabeled Chemicals, Inc., Saint Louis, USA) during a 9 h period in a sealed incubation system at 28°C, and with a dose of 1.6 μl of the [U-^14^C] protein hydrolysate per ml of seawater (Morais et al., 2004a). The incubation system consisted of an aquarium with controlled temperature and an incubation bottle connected to a KOH trap to capture radiolabeled ^14^CO_2_ (Morais et al., 2004a). This process was carried out twice, in order to have an *Artemia* labeled batch for the first three trials, and a second one for the last two trials. This protocol ensured that similar prey was offered to all fish independently of the 24 h cycle.

To evaluate the amount of label incorporated by *Artemia*, *Artemia* metanauplii were washed several times before each trial and counted. Samples (*n*=4, 3 ml per sample) were also taken to measure the incorporated radiolabel as described in Morais et al. (2004a). Seawater from the beaker containing the radiolabeled *Artemia* was sampled in order to correct the ^14^C present in the incubation seawater (*n*=4, 3 ml per sample).

### Senegalese sole rearing

Post-larvae were obtained from Sea8 (Póvoa de Varzim, Portugal) at 20 days post-hatching (dph), with an average dry weight (DW) of 1.51±0.10 mg. Post-larvae were kept in a recirculation system at the Centre of Marine Sciences (University of Algarve, Faro, Portugal) in three 3-liter flat bottom trays with an initial density of 80 post-larvae L^−1^ until metabolism assays were performed. Photoperiod was 12 h light:12 h dark (lights turned on and off at 9:00 h and 21:00 h, respectively), salinity was 34±1.3 g l^−1^ and temperature 19.6±0.9°C. Up to 33 dph, post-larvae were fed to satiation four times daily with *Artemia* metanauplii enriched with DHA Selco (INVE Aquaculture, Belgium). At 33 dph, 20 post-larvae tray^−1^ were sampled, rinsed in distilled water and individually freeze-dried to dry weight determination.

### Radiolabel measurements

Post-larvae and *Artemia* dissolved tissues were prepared for scintillation counting by adding 4 and 16 ml of scintillation cocktail (Ultima Gold XR, PerkinElmer, USA), respectively. 16 and 14 ml of scintillation cocktail were also added to the incubation water and metabolic trap vials, respectively. Samples were counted for radioactivity (DPM, disintegrations per minute) in a liquid scintillation counter (Tri-Carb 2910TR, PerkinElmer, USA). All counts were corrected for quench and lumex. Post-larvae feed intake after one single meal (FI, % DW) was individually calculated as described by Conceição et al. (1998):
(1)
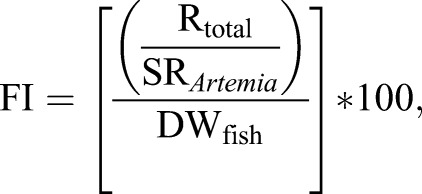


where, R_total_ is the sum of the radioactivity in the incubation water, in the CO_2_ trap and in fish (DPM), SR_*Artemia*_ is the specific radioactivity in *Artemia* samples (DPM/mg *Artemia* DW), and DW_fish_ is the fish dry weight (mg).

Protein utilization was individually determined based on protein digestibility, retention efficiency and catabolism fraction. These estimates were determined as follows:
(2)
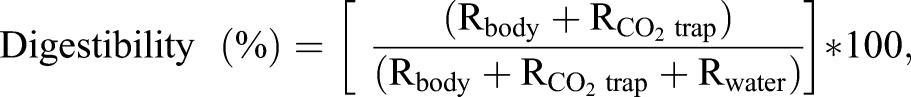

(3)
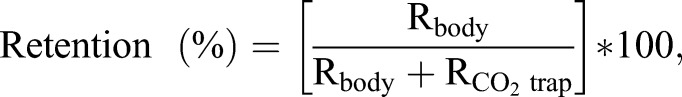

(4)
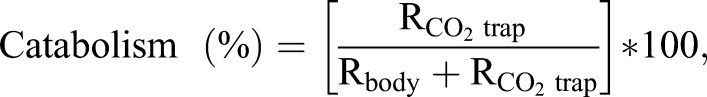


where R_body_ is the total radioactivity in fish body (DPM), R_CO_2__ trap is the total radioactivity per CO_2_ trap (DPM), and R_water_ is the total radioactivity in the incubation seawater (DPM).

### Statistical analysis

Fish that did not ingest any *Artemia* during the 30 min feeding period were excluded from the analysis; 09:00 h (*n*=10), 15:00 h (*n*=11), 21:00 h (*n*=12), 03:00 h (*n*=12) and 09:00 h+1 d (*n*=9). All percentage data were arcsine square root-transformed prior to analysis. In order to examine differences in feed intake and protein metabolism between treatments, one-way ANOVA was performed after assessing equality of variances by a Levene's test. In cases where significant differences were found (*P*<0.05), a Tukey's multiple comparison test was carried out to determine which specific trial differed significantly. All statistical analyses were performed with SPSS 15.0 software (IBM, New York, USA) and the results are given as means and standard deviations (s.d.).
